# A novel promoter controls Cyp19a1 gene expression in mouse adipose tissue

**DOI:** 10.1186/1477-7827-7-37

**Published:** 2009-04-24

**Authors:** Hong Zhao, Joy Innes, David C Brooks, Scott Reierstad, Mehmet B Yilmaz, Zhihong Lin, Serdar E Bulun

**Affiliations:** 1 Department of Obstetrics and Gynecology, Feinberg School of Medicine, Northwestern University, Chicago, Illinois 60611, USA

## Abstract

**Background:**

Aromatase, the key enzyme in estrogen biosynthesis, is encoded by the Cyp19a1 gene. Thus far, 3 unique untranslated first exons associated with distinct promoters in the mouse Cyp19a1 gene have been described (brain, ovary, and testis-specific). It remains unknown whether aromatase is expressed in other mouse tissues via novel and tissue-specific promoters.

**Methods:**

Real-time PCR was used to examine the aromatase expression levels in various C57BL/6 mouse tissues. 5'-rapid amplification of cDNA ends (5'-RACE) was used to determine the transcriptional start sites of Cyp19a1 transcripts. Promoter activity was measured using serial deletion mutants of DNA fused to the luciferase reporter gene. Primary mouse adipose fibroblasts were isolated and cultured from 16-week-old mouse gonadal fat pads.

**Results:**

We systematically analyzed Cyp19a1 expression in a large number of mouse tissues, and demonstrated for the first time that aromatase was expressed in the male but not female gonadal fat pad. Subcutaneous and brown adipose tissue did not contain detectable Cyp19a1 mRNA. We used 5'-RACE to clone a novel gonadal fat-specific untranslated first exon, which is spliced onto a common junction 15 bp upstream of the translation start site. This adipose-specific first exon was mapped to approximately 75 kb upstream of the translation start site. Transfection of luciferase reporter gene plasmids containing the promoter region upstream of the adipose-specific first exon into murine 3T3-L1 adipose fibroblasts demonstrated significant basal promoter activity conferred primarily by the sequence located at -343/-1 bp. Dexamethasone significantly induced activity of this adipose-specific promoter region. Adipose-specific Cyp19a1 mRNA was expressed in primary mouse adipose fibroblasts and significantly induced by dexamethasone alone or serum plus dexamethasone.

**Conclusion:**

Taken together, this research identified a novel, adipose-specific first exon of Cyp19a1 and its hormonally regulated promoter region in male murine gonadal fat. These results expand the known 5'-regulatory region of the murine Cyp19a1 gene to 75 kb upstream of the translation start site. Cyp19a1 expression in mouse adipose tissue may play an important role in reproductive biology and lipid metabolism.

## Background

Aromatase cytochrome P450 is the key enzyme for estrogen biosynthesis that converts androstenedione and testosterone to estrone and 17β-estradiol, respectively [1]. The biologically active estrogen, 17β-estradiol, functions primarily via binding to its receptors, estrogen receptor-α [2-4] and estrogen receptor-β [5]. Beyond its essential role in reproductive function [6], estrogen is also involved in vascular biology [7,8], lipid and carbohydrate metabolism [9,10], bone mineralization [11], and cognitive and other brain-related functions [1,12]. Estrogen is also essential for the initial development and further growth of a number of benign and malignant hormone-dependent disorders [6].

Aromatase is encoded by the *CYP19A1 *gene in humans, which spans approximately 123 kb on chromosome 15q21.2. The ATG translational start site is located in coding exon II, and the coding region of aromatase protein is found within 30 kb of the 3'-end and contains nine exons (II-X) [6]. The 93-kb 5'-flanking region upstream of the coding region contains a number of alternative untranslated first exons, the expression of which is driven by multiple tissue-specific promoters [13]. These promoters differentially regulate expression of aromatase in gonads, adipose tissue, bone, brain, skin, fetal liver, and placenta [14]. Thus far, 10 alternative tissue-specific first exons have been found in the human, including exon I.1, I.2 and I.2a in placenta, I.4 in adipose tissue and skin, I.5 in fetal tissues, I.f in brain, I.7 in endothelial cells, I.6 in bone, I.3 in adipose tissue, and PII in gonads. The most proximal promoter, PII, and 2 other proximal promoters, I.3 and I.6, are within the 1-kb region upstream of the ATG translational start site, whereas promoter I.4 is located 73 kb upstream of exon II. The most distally located promoter, I.1, is found approximately 93 kb upstream of the coding region. All of these 5'-untranslated first exons are spliced onto a common junction located 38 bp upstream of the ATG translational start site. Consequently, the aromatase protein is the same regardless of the splicing pattern [15].

In mice, aromatase is encoded by a single gene, *Cyp19a1*, located on chromosome 9. Similar to humans, the ATG translation start site lies in coding exon II and the coding region of aromatase protein is found in the downstream 29-kb portion of the gene and contains 9 exons (II-X) [16]. In contrast to the human gene, only 3 tissue-specific untranslated first exons of the mouse *Cyp19a1 *gene have been reported, including an ovary-specific first exon (Eov), a testis-specific first exon (Etes), and a brain-specific first exon (Ebr). To generate mouse tissue-specific aromatase transcripts, a tissue-specific first exon is spliced onto a common coding region as a result of the activation of its upstream promoters, which regulate aromatase expression in the ovary, testis, or brain. The mouse ovary- and brain-specific first exons share 100% and 93% homology with the human exons PII and I.f, whereas the recently discovered testis-specific first exon is unique to the mouse. According to a previous study, the entire mouse *Cyp19a1 *locus is approximately 60 kb. The 3 untranslated first exons and their flanking promoter regions span more than 30 kb, whereas the 9 coding exons are restricted to about 29 kb. Promoters for Ebr and Etes are located about 31 kb and 10 kb upstream of the translation start site. As in other species, the promoter for Eov is the most proximal promoter, located 121 bp upstream of the translation start site [17,18].

In the human, extragonadal aromatase expression plays a key role in estrogen production, especially in men and in postmenopausal women, in whom ovarian aromatase expression ceases after menopause [6,19,20]. In particular, skin and adipose fibroblasts express physiologically significant levels of aromatase to produce sufficient quantities of estrogen, which may prevent bone loss in both sexes or contribute to endometrial hyperplasia or cancer in women [21,22]. Promoter I.4 is primarily responsible for regulating aromatase expression in adipose tissue and skin in humans [23]. Aromatase expression, however, has not been reported in mouse adipose or skin tissue. Thus, we were particularly interested in determining whether aromatase is expressed in mouse extragonadal tissues, including fat or skin, and whether a novel promoter comparable to the human promoter I.4 regulates peripheral aromatase expression in mice.

## Methods

### Animals

Animals were housed according to the National Institutes of Health Guide for the Care and Use of Laboratory Animals. All procedures were approved by the Northwestern University Animal Care and Use Committee. All tissues were harvested from mice with a C57BL/6J background. Mice were maintained on a 14-hour light:10-hour dark cycle with standard chow (7912; Harlan Teklad, Madison, WI) and water *ad libitum*.

### Quantitative real-time RT-PCR

Total RNA from various mouse tissues was extracted at 10 and 16 weeks of age using TRIzol reagent according to the manufacturer's instructions (Sigma, St. Louis, Missouri, USA). cDNA was synthesized using oligo (dT) primers with superscript™ III first-strand kit as recommended by the supplier (Invitrogen, Carlsbad, California, USA). Real-time PCR was performed with the Power SYBR^® ^green PCR kit according to the manufacturer's instructions (Applied Biosystems, Foster City, California, USA) in an ABI 7900 HT fast real-time PCR system (Applied Biosystems). The primers used were as follows:

*Cyp19a1 *forward: 5'-CTG CAG ACA CTA CTA CTA CA-3'

*Cyp19a1 *reverse: 5'-ATC CGA GTC ACT GCT CTC AG-3'

GAPDH forward: 5'-TGT TAC CAA CTG GGA CGA CA-3'

GAPDH reverse: 5'-GGG GTG TTG AAG GTC TCA AA-3'

To generate external standard curves for each run, mouse aromatase and GAPDH cDNAs were amplified from ovarian tissue and cloned into the pCR^®^II-TOPO plasmid (Invitrogen). The following standard primers were used:

standard *Cyp19a1 *forward: 5'-GCT GAA CCC CAT GCA GTA TAA-3'

standard *Cyp19a1 *reverse: 5'-AGC CAA AAG GCT GAA AGT ACC-3'

standard GAPDH forward: 5'-CCG CAT CTT CTT GTG CAG T-3'

standard GAPDH reverse: 5'-TCC ACC ACC CTG TTG CTG TA-3'

Different concentrations (10^-1 ^to 10^-12 ^μg DNA/μl) of plasmid DNA were amplified with the real-time PCR primers using the Power SYBR^® ^green PCR kit as standard curves. Copy numbers in various tissues were calculated relative to the amount of total RNA used. The ratio of copy numbers of *Cyp19a1 *(× 10^7^) to copy numbers of GAPDH was calculated as *Cyp19a1 *mRNA levels. Pituitary RNA was analyzed from pools of 5 animals. Each pool was measured in at least 3 independent reverse transcription (RT) reactions, and the mean and SEM were calculated. Other tissues were measured as individual samples. Real-time PCR cycler conditions were 50°C for 2 minutes; 95°C for 10 minutes; and 40 cycles of 95°C for 15 seconds and 60°C for 1 minute.

### 5'-rapid amplification of cDNA ends (5'-RACE)

**Figure 3:**
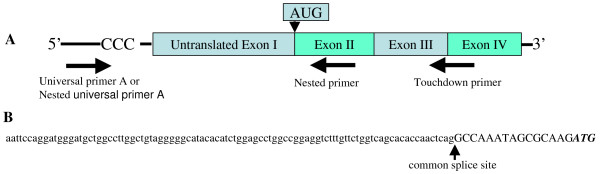
**Isolation of a *Cyp19a1 *transcript with an untranslated adipose-specific first exon in male mouse gonadal fat**. (A) Schematic representation of relationship of gene-specific primers to *Cyp19a1 *cDNA template for 5'-RACE. (B) Nucleotide sequence of the 5' region of *Cyp19a1 *cDNA isolated from male mouse gonadal fat. Arrows indicate the 5'-boundary of region encoded by exon II of the *Cyp19a1 *gene. The lower case letters show the unique DNA sequence of the adipose-specific first exon. ATG indicates the translational start site.

To determine the transcriptional start sites of *Cyp19a1 *transcripts from various tissues, 5'-RACE was performed using the SMART™ RACE cDNA amplification kit (Clontech, Mountain, California, USA). One microgram of total RNA from each tissue (ovary, testis, hypothalamus, or male gonadal fat) underwent RT with a modified oligo (dT) primer. After PowerScript RT reached the end of the mRNA template, it added several dC residues. The SMART II™ A oligonucleotide annealed to the tail of the cDNA and served as an extended template for PowerScript RT. The primary touchdown PCR reverse primer (5'-GAC TCT CAT GAA TTC TCC ATA CAT CT-3', Figure 3A), binding to exon 3 and 4 coding sequence, was combined with the universal primer A mix provided with the kit. If the primary PCR reaction failed to give the distinct bands of interest, a secondary, or "nested" PCR reaction was performed with the reverse nested primer (5'-AAT GAG GGG CCC AAT TCC CAG A-3', Figure 3A), binding to exon 2 coding region, combined with the nested universal primer A. RACE products were cloned into the pCR^®^-TOPO TA cloning vector and subsequently sequenced.

### Exon-specific RT-PCR and real-time RT-PCR amplification

Total RNA was extracted from various mouse tissues and treated with the TURBO DNase following the standard protocol (Applied Biosystems). The treated RNA was then reverse transcribed using oligo (dT) primers with superscript™ III first-strand RT kit according to the instructions of the manufacturer (Invitrogen Corp.). To amplify the transcript variants in male gonadal adipose tissue, ovary, testis, and brain (Tad, Tov, Ttes, and Tbr), the sequence information of RACE products was used to design forward primers that bound the 5' untranslated regions (5' UTR) of these transcripts. The reverse primers were located in coding exon II. The following primer pairs were used:

Tad forward: 5'-GTT CTG GTC AGC ACA CCA ACT-3'

Tad reverse: 5'-AGA CGA GCT CTC ACA ATT CCA-3'

Tov forward: 5'-CAC CAC TGC TTT CTT CCC ATA-3'

Tov reverse: 5'-AGA CGA GCT CTC ACA ATT CCA-3'

Ttes forward: 5'-GGT TAC CAT GGG GAA CGA A-3'

Ttes reverse: 5'-ATT CCC AGA CAG TAG CCA GGA-3'

Tbr forward: 5'-CTG CGC ATC ATT AGC AAA ACT-3'

Tbr reverse: 5'-AGA CGA GCT CTC ACA ATT CCA-3'

The PCR cycler conditions were 95°C for 4 minutes; 35 cycles of 94°C for 30 seconds, 60°C for 30 seconds, and 72°C for 1 minute; and a final extension of 72°C for 9 minutes.

Real-time PCR cycler conditions were 50°C for 2 minutes; 95°C for 10 minutes; and 40 cycles of 95°C for 15 seconds and 60°C for 1 minute.

### Plasmids, transfections, and luciferase assays

The potential promoter regions of an adipose-specific transcript (343 bp and 1637 bp upstream of transcriptional start site) were amplified by PCR. The primers were introduced at the restriction enzyme KpnI and BglII sites. The PCR fragments were then digested and subcloned into a pGL4.10 [*luc2*] luciferase vector. Briefly, the promoter region was cloned into the location between a synthetic poly (A) signal/transcriptional pause site and the luc2 gene of the pGL4.10 [*luc2*] vector (Promega, Madison, Wisconsin, USA). All constructs were reconfirmed by sequencing. The primers amplifying the promoter region were:

-343 forward: 5'-ATA GGT ACC CCA CGA TAA GGG ACT TGA TGA-3'

-1637 forward: 5'-ATA GGT ACC TCT CAT CGT CTG ATG TTC TGG-3'

-343 and -1637 reverse: 5'-ATT AGA TCT ACG GAA TCC CCA CCC TGC CTC A-3'

Mouse 3T3 L1 cells were transfected with each of the promoter constructs using Lipofectamine™ 2000 transfection reagent (Invitrogen) according to the manufacturer's protocol. Reporter plasmid (4 μg) and a pGL4.74 [hRluc] internal control (0.4 μg) were transfected per well of 6-well plate for 24 hours. Following serum starvation, cells were maintained in DMEM supplemented with 10% fetal bovine serum (FBS) in the absence or presence of 250 nM dexamethasone for 24 hours, and total protein lysate was collected and assayed for luciferase activity.

### Primary mouse adipose fibroblast (MAF) culture and hormone treatment

Mouse gonadal fat pads were harvested from 16-week-old mice. Isolation and culture of primary MAFs were performed according to the modified previous protocol for culturing human breast adipose fibroblasts [24]. In brief, adipose tissues were minced and digested with collagenase B (1 mg/ml) at 37°C for 30 minutes. Single-cell suspensions were prepared by filtration through a 75-μm sieve. Fresh cells were suspended in DMEM/F-12 containing 10% FBS. Twelve to 24 hours after fibroblast attachment, culture medium was changed at 48-hour intervals. Cells were grown to confluence and placed in serum-free medium for 16 hours. MAFs were incubated in serum-free DMEM/F-12 medium or DMEM/F-12 supplemented with 10% FBS in the absence or presence of 250 nM dexamethasone or 500 nM Dibutyryl cAMP (Bt2cAMP) plus 100 μM phorbol diacetate (PDA) for 24 hours. Cell extracts were prepared as previously described for real-time RT-PCR [25].

### Statistical analysis

Results are expressed as mean ± SEM. Statistically significant differences at p < 0.05 were determined by 1-way ANOVA followed by t-test.

## Results

### Expression of the *Cyp19a1 *gene in male mouse gonadal fat

**Figure 1:**
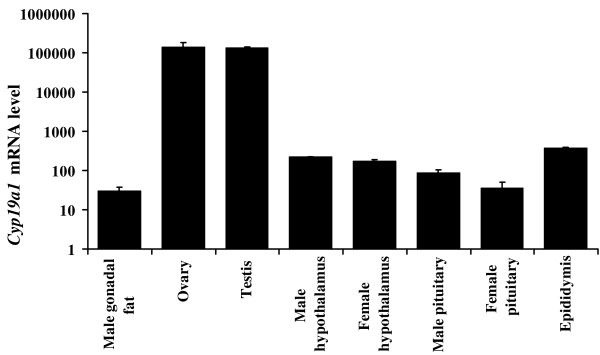
**The level of *Cyp19a1 *mRNA in 10-week-old mouse tissues measured by real-time RT-PCR**. *Cyp19a1 *and GAPDH cDNAs from mouse ovary were cloned into pCR^®^II-TOPO plasmid vector. Different concentrations (10^-1 ^to 10^-12 ^μg DNA/μl) of plasmid DNAs containing *Cyp19a1 *or GAPDH sequence were amplified with primers for *Cyp19a1 *or GAPDH as the external standard curves. The ratio of copy numbers of *Cyp19a1 *(× 10^7^) to copy numbers of GAPDH was calculated as *Cyp19a1 *mRNA levels. Pituitary was analyzed from pools of 5 animals. Data are expressed as mean ± SE with n = 5 mice in each group.

To determine *Cyp19a1 *mRNA expression in mouse tissues, 10-week-old mice were euthanized and multiple tissues were collected and analyzed by quantitative real-time RT-PCR. After 40 cycles of PCR, if the Ct value is underdetermined, we considered *Cyp19a1 *mRNA below the level of detection. As expected, aromatase mRNA was detected in the testis and epididymis in males, the ovary in females, and the hypothalamus and pituitary in both sexes (Figure 1). Neither male nor female mice had detectable *Cyp19a1 *mRNA in lung, liver, kidney, spleen, bone, stomach, intestine, heart, aorta, muscle, or skin. *Cyp19a1 *mRNA was also below the level of detection from the uterus, mammary gland, and vas deferens (data not shown). A systematic analysis of adipose tissues from various body sites was also performed. We demonstrated for the first time that aromatase was expressed in the male but not female gonadal fat pad. Subcutaneous and brown adipose tissue did not contain detectable *Cyp19a1 *mRNA.

*Cyp19a1 *mRNA levels in various male and female mouse tissues are illustrated in Figure 1. The highest levels of *Cyp19a1 *mRNA were found in the ovary and testis, followed by the hypothalamus and epididymis, whereas the lowest levels were detected in the male gonadal fat and pituitary.

**Figure 2:**
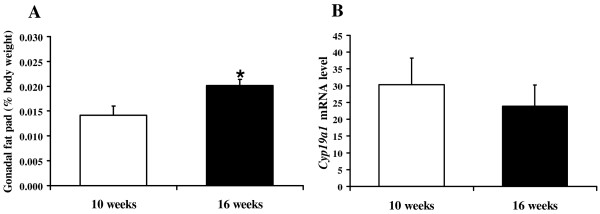
***Cyp19a1 *gene expression in 10- and 16-week-old male mouse gonadal fat**. (A) Gonadal fat pad weight as percentage of body weight. (B) *Cyp19a1 *mRNA levels in male mouse gonadal fat were measured by real-time RT-PCR. Data are expressed as mean ± SE with n = 5–8 mice in each group. *p < 0.05 for 16-week-old mice vs. 10-week-old mice.

We next determined whether *Cyp19a1 *expression in male gonadal fat varied with age or amount of tissue. While gonadal fat pad weight increased by 43% in 16-week-old mice compared with 10-week-old mice (Figure 2A), Cyp19a1 mRNA levels were not significantly different (Figure 2B).

### Isolation of a novel untranslated gonadal adipose tissue-specific first exon of the *Cyp19a1 *gene

To determine whether the transcription initiation site and the untranslated first exon of *Cyp19a1 *transcripts in gonadal fat were different than those of previously published sequences, we performed 5'-RACE (Figure 3A). Nine 5'-RACE clones from male gonadal fat (n = 3) were isolated and identified as *Cyp19a1 *transcripts (Ensembl Transcript ID: ENSMUST00000034811). The nucleotide sequence of the 5' region of aromatase adipose-specific transcript (Tad) is shown in Figure 3B (GenBank accession number: EU252014, date of accession: Nov. 1, 2009). The untranslated first exon in gonadal fat was 93 bp and was located 74763 bp upstream of the translational start site. The 5' UTRs of the 9 isolated clones were identical and were not similar to any previously known *Cyp19a1 *transcript sequence.

5'-RACE clones from 3 other tissues (ovary, testis, and brain) were analyzed as controls. Five clones from the ovary had identical transcriptional start sites (position 221 of EMBL/GenBank accession number D67046), and the sequences of the 5' UTRs of the ovarian transcripts (Tov) were identical to ovarian *Cyp19a1 *cDNA described previously [18]. *Cyp19a1 *transcripts from 8 5'-RACE clones were identified as brain aromatase cDNA (Tbr) [17]. The transcription start site in male mice was at position 382 of EMBL/GenBank accession number D67045, which was 47 nucleotides upstream from that described previously [17]. The transcription start site in female mice was at position 444 of EMBL/GenBank accession number D67045, 15 nucleotides downstream from that described previously (17). The transcription start site of the testis-specific transcript (Ttes) was located at position 1589 of EMBL accession number AJ437576, which was 18 nucleotides downstream from that described by Vanselow et al. [16]. The small discrepancy between our results and previous published data with respect to the presumed transcription start sites of brain- and testis-specific *Cyp19a1 *mRNAs may be due to a range of possible initiation sites within a confined region or slightly different techniques used in various laboratories.

### Expression of mouse *Cyp19a1 *adipose-specific transcript in various tissues

**Figure 4:**
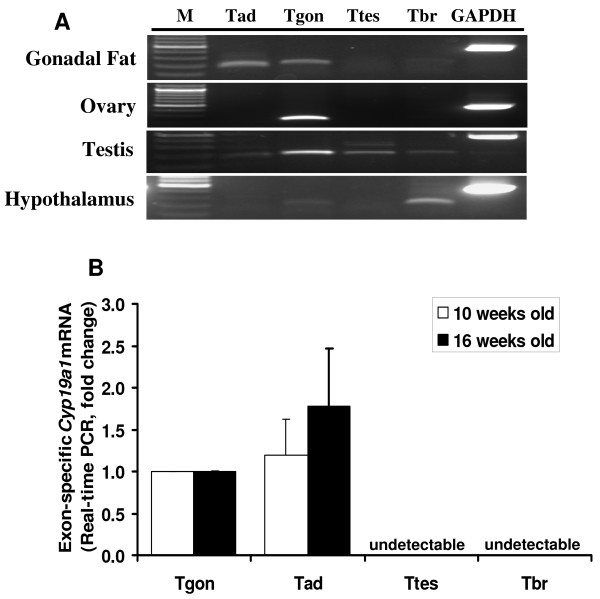
**Expression of adipose-specific transcripts of the *Cyp19a1 *gene in different mouse tissues using RT-PCR (A) and in gonadal fat using real-time RT-PCR(B)**. Tissue-specific primer pairs were used. Each forward primer was located in the tissue-specific first exon. Reverse primers were located in coding exon II. Tad: transcript in adipose tissue, Tgon: transcript in gonad, Ttes: transcript in testis, Tbr: transcript in brain, M: DNA ladder.

To determine the distribution of Tad, Tov, Ttes, and Tbr in male gonadal fat, ovary, testes, and hypothalamus, exon-specific RT-PCR analysis of RNAs was performed using 5'-UTR-specific forward primers and reverse primers located in coding exon II. Tad and Tov were expressed in male gonadal fat (Figure 4A). Those results were confirmed by real-time RT-PCR in 10-week-old and 16-week-old mouse gonadal fat (Figure 4B). Tbr and Tov were found in the hypothalamus. Tov was the only *Cyp19a1 *transcript expressed in the ovary. All 4 transcripts were found in the testis, though Tov was the major transcript. Thus, we suggested renaming the previously described Tov as gonadal-specific transcript (Tgon).

### Male gonadal adipose-specific promoter activity

**Figure 5:**
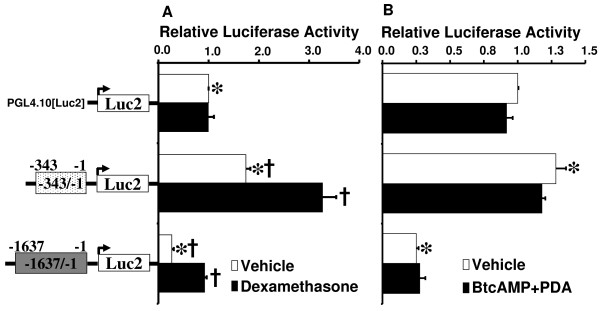
**Regulation of the adipose-specific promoter of the *Cyp19a1 *gene by dexamethasone (A) and Bt2cAMP plus PDA (B)**. Luciferase plasmids containing the 5'-flanking promoter region of the mouse *Cyp19a1 *gene in gonadal fat (-343/-1 bp and -1637/-1 bp) were transfected into murine 3T3 L1 cells for 24 hours. pGL4.74 [hRluc] *Renilla *was used as an internal control for transfection efficiency. Promoter activity was normalized to pGL4.74 [hRluc] *Renilla *and was represented as the average of data from triplicate replicates; the empty luciferase vector PGL4.10 [*luc2*] without treatment was arbitrarily assigned a unit of 1, and results are expressed as multiples of the PGL4.10 [*luc2*] vector. All results are given as mean ± SE from at least 3 independent experiments performed in triplicate. *p < 0.05 for vs. the empty luciferase vector PGL4.10 [*luc2*] without treatment. †p < 0.05 for vs. vehicle treatment.

In order to characterize promoter activity of the newly identified male gonadal fat-specific transcript, we generated 2 luciferase reporter constructs containing -343/-1 and -1637/-1 bp of the 5' sequence and assessed their activity in luciferase reporter assays. As compared with the empty control vector, luciferase activity was increased by 1.7 fold in cells transfected with the -343/-1 construct, and decreased by 3.7 fold in cells transfected with the -1637/-1 construct (Figure 5A). This result indicated that basal promoter activity is conferred by a sequence located within -343/-1 bp and there may be a negative regulatory sequence within the -1637/-343 bp region of the promoter. Luciferase activity of the empty vector was not altered following dexamethasone treatment, whereas activity of the -343/-1 promoter increased by 1.6 fold after treatment with dexamethasone compared with vehicle. In the presence of dexamethasone, luciferase activity of -1637/-1 increased by 3.4 fold as compared with vehicle treatment. Both basal and dexamethasone-stimulated promoter activity levels were significantly diminished in -1637/-1 compared with the -343/-1 sequence. Although dexamethasone response was preserved in both, this is suggestive of a general silencer-type element in the -1637/-343 region, which does not eliminate glucocorticoid action. Bt2cAMP plus PDA did not induce activity of the gonadal adipose-specific *Cyp19a1 *promoter (Figure 5B).

### Dexamethasone plus fetal bovine serum stimulated *Cyp19a1 *mRNA expression in primary MAFs

**Figure 6:**
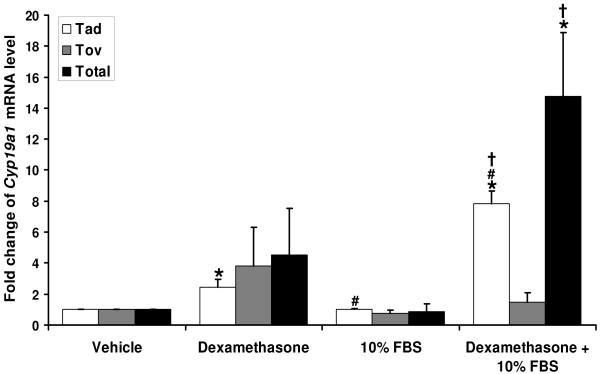
**Dexamethasone or dexamethasone plus FBS induced adipose-specific *Cyp19a1 *mRNA expression in primary MAF**. Following overnight serum starvation, cells were incubated in serum-free DMEM/F-12 medium or DMEM/F-12 supplemented with 10% FBS in the absence or presence of 250 nM dexamethasone for 24 hours. Adipose-specific, ovarian-specific or total C *yp19a1 *mRNA levels were analyzed by real-time RT-PCR. *p < 0.05 for vs. vehicle treatment. #p < 0.05 for vs. Dexamethasone treatment. †p < 0.05 for vs. FBS treatment.

The literature indicates that hormonal treatments with broad actions such as PKA stimulators (cAMP analogs), PKC stimulators (phorbol esters), glucocorticoids and serum, alone and in combination, regulated aromatase expression in human adipose fibroblasts via different promoters[26,27]. To determine aromatase expression and regulation in MAFs, mouse gonadal fat pads were harvested and primary MAFs were cultured. Based on our results regarding the activity of the adipose tissue promoter (Figure 5), we treated these cells with vehicle, dexamethasone, FBS, or dexamethasone plus FBS. We quantitated the levels of various promoter-specific *Cyp19a1 *mRNA expressed using exon-specific real-time RT-PCR. Adipose-specific *Cyp19a1 *mRNA levels were significantly increased by 2.5 fold upon dexamethasone treatment and further significantly increased to 7.8 fold following dexamethasone plus FBS treatment. Dexamethasone, FBS, or dexamethasone plus FBS treatment did not significantly alter ovarian-specific *Cyp19a1 *mRNA levels. Although total *Cyp19a1 *mRNA levels were increased by 4.5 fold following dexamethasone treatment as compared to vehicle treatment, statistically this result did not rise above the level of significance. FBS alone had no effect on *Cyp19a1 *expression, but significantly augmented the effect of dexamethasone to cause a 15-fold increase in *Cyp19a1 *expression (Figure 6).

## Discussion

In humans and mice, *Cyp19a1 *gene expression is controlled by various tissue-specific promoters that drive the transcription of untranslated tissue-specific first exons together with the common coding exons II-X to generate aromatase [6,13]. Thus far, 2 adipose tissue-specific first exons in humans have been identified, exons I.4 and I.3, located 73 kb and within 1 kb upstream of the translational start site, respectively. Here, we demonstrated for the first time the presence of an adipose tissue-specific untranslated *Cyp19a1 *first exon in male, but not female, mouse gonadal fat.

**Figure 7:**
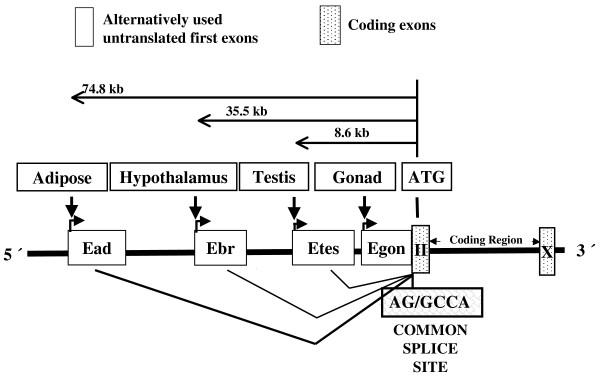
**Schematic of the alternatively used untranslated first exons, coding exons, and common splice site of the mouse *Cyp19a1 *gene**.

The mouse *Cyp19a1 *gene, including 3 unique untranslated first exons, is expressed in ovary, testis, and brain [16]. A previous study showed that the brain-specific first exon is the most distally located first exon in mice, 31 kb upstream of the translational start site [17]. Here, we found that the gonadal-fat specific first exon is located 75 kb upstream of the translational start site (Figure 7). We assumed some similarities between human exon I.4 and the mouse adipose-specific first exon (Ead). However, analysis of the sequence of exon I.4 and Ead showed no significant similarity, nor did the Ead sequence show similarities to any other previously reported exons in humans or mice using the BLAST (Basic Local Alignment Search Tool). Thus, our results extended the *Cyp19a1 *5' UTR from 31 kb up to 75 kb upstream of the first coding exon. Based on our data, the mouse *Cyp19a1 *gene now includes at least 104 kb of genomic sequence on chromosome 9.

This newly identified Tad was expressed in male gonadal fat and testis, and was not found in ovary or brain. It has been shown that Tgon is the principle transcript variant in the mature ovary [18]. A previous study also showed that Tgon is expressed in the testis and brain [16], and we found that Tgon was also expressed in adipose tissue. Tbr was only expressed in brain and testis, which was not consistent with a previous study which also described ovarian expression of Tbr. As shown in the previous study [16], Ttes was uniquely expressed in testis, but we also found that Tgon was the major *Cyp19a1 *transcript in testis.

*CYP19A1 *expression in premenopausal women is mainly found in ovarian follicles, and estrogen functions as a circulating hormone on distal target tissues. Conversely, local estrogen synthesis in extragonadal tissues, such as adipose tissue, plays a more predominant role in men and postmenopausal women [28,29]. Regional variations in *CYP19A1 *expression in subcutaneous adipose tissue have been observed in humans, and its expression in buttocks and thighs is 2- to 3-fold greater than that in subcutaneous abdominal tissue and breast tissue [30]. In the present study, we showed regional variations of *Cyp19a1 *expression in mice. *Cyp19a1 *was expressed in male, but not female, mouse gonadal fat, and there was no *Cyp19a1 *expression in subcutaneous fat or brown adipose tissue in either sex.

The *CYP19A1 *transcripts in human breast adipose tissue contain exon I.4 followed by exon 1.3 and II. In adipose stromal cells, the transcription of I.3 and II is stimulated by cAMP or prostaglandin E2. Promoter I.4 contains the interferon γ activation element followed by a glucocorticoid response element that is a SP1-binding site, and I.4 expression is stimulated by class I cytokines and TNFα through the JAK1/STAT3 pathway [1]. We found that *Cyp19a1 *promoter activity in male mouse gonadal fat was induced by dexamethasone, and did not change after Bt2cAMP plus PDA treatment. Adipose-specific *Cyp19a1 *mRNA expression in primary MAF was increased by dexamethasone and augmented by FBS. This response is similar to that seen in human promoter I.4 activation, which is enhanced by binding of glucocorticoid receptors to glucocorticoid response elements. We also performed a TF search for cis-regulating elements in the new adipose promoter and found three half glucocorticoid response elements(GREs) located at -139/-134, -163/-159,-316/-311bp. Thus, it is likely that this new mouse adipose-specific aromatase promoter has a similar regulatory profile to the human adipose/skin promoter I.4, which has binding sites for glucocorticiod receptor. Our results from both Ead luciferase reporter assay and primary cultured mouse adipose fibroblasts support this notion. The promoter activity of the *Cyp19a1 *transcript in male mouse gonadal fat may also be induced through JAK1/STAT3 pathway. Thus, cytokines in serum may function synergistically with glucocorticoid to activate this promoter [31].

Estrogens play an important role in the maintenance of lipid homeostasis [32]. The aromatase knockout (ArKO) mouse cannot synthesize endogenous estrogens, and circulating estradiol levels are at the limit of detection. ArKO mice develop an age-dependent increase in intra-abdominal adiposity. The increase in adipose tissue is associated with hyperplasia and hypertrophy of the adipocytes with a simultaneous decrease in lean mass and development of hypercholesterolemia, hyperleptinemia, and insulin resistance [9,33]. Total body fat is also increased in 16-week-old mice in estrogen receptor α and/or β knockout mice [34-36]. Aromatase-deficient patients show a similar phenotype characterized by impaired lipid and insulin metabolism [14]. In our study, we found that *Cyp19a1 *was expressed in male mouse adipose tissue. In addition to circulating estradiol, locally produced estradiol in male mouse gonadal fat may play an important physiologic role in regulating lipid metabolism. Moreover, estrogen produced in gonadal fat may exert local physiologic effects on testicular maturation.

In summary, we have shown *Cyp19a1 *expression in male mouse gonadal fat, isolated an adipose-specific untranslated first exon, and demonstrated dexamethasone-regulated promoter activity of the adipose-specific *Cyp19a1 *transcript. Together with other studies [16-18], these results suggest that in mice, at least 4 different promoters actively regulate *Cyp19a1 *expression in adipose tissue, gonads, and brain. These results expanded the 5'-regulatory region of the murine *Cyp19a1 *gene to 75 kb upstream of the translation start site (Figure 7). Further studies are necessary to identify the cis-regulatory elements and transcriptional factors that regulate the novel gonadal fat-specific *Cyp19a1 *promoter.

## Competing interests

The authors declare that they have no competing interests.

## Authors' contributions

HZ carried out all experiments with the following coauthors helps. JI participated in real-time PCR, 5'-RACE and primary mouse adipose fibroblast culture. DB has been involved in drafting the manuscript and primary mouse adipose fibroblast culture. SR supported the experiment of real-time PCR. MY supported the experiment of 5'-RACE. ZL supported the luciferase assays. SB conceived of the study, and participated in its design and coordination and helped to draft the manuscript. All authors read and approved the final manuscript.
